# SimpleMicrobiome: An integrated web-based platform for streamlined microbiome data analysis and visualization

**DOI:** 10.71150/jm.2606011

**Published:** 2026-09-18

**Authors:** Seong-In Na, Juhee Kim, So-Yeon Kim, Jin Park, Yong-Joon Cho

**Affiliations:** 1 Multidimensional Genomics Research Center, Kangwon National University, Chuncheon 24341, Republic of Korea; 2 Department of Molecular Bioscience, College of Biomedical Science, Kangwon National University, Chuncheon 24341, Republic of Korea; 3 Department of Dermatology, College of Medicine, Jeonbuk National University, Jeonju 54907, Republic of Korea; 4 Research Institute of Clinical Medicine of Jeonbuk National University, Biomedical Research Institute of Jeonbuk National University Hospital, Jeonju 54907, Republic of Korea

**Keywords:** microbiome analysis, web-based platform, differential abundance, diversity analysis, network analysis, workflow

## Abstract

Microbiome studies require multiple analytical steps after initial sequence processing. These steps commonly include data harmonization, preprocessing, taxonomic profiling, diversity analysis, differential abundance testing, predictive modeling, network inference, and preparation of publication-ready outputs. Although robust packages are available for many of these tasks, routine use often depends on command-line workflows, repeated data reformatting, and method-specific scripting. These requirements can limit accessibility for experimental researchers and complicate consistent analysis across interdisciplinary teams. We developed SimpleMicrobiome, a web-based R Shiny platform that integrates established microbiome analysis methods into a single interactive downstream workflow. The application accepts standard abundance, taxonomy, and metadata tables, supports interactive preprocessing and sample filtering, and provides modules for taxa profile visualization, alpha and beta diversity analysis, ANCOM-BC2 and MaAsLin2 differential abundance testing, Random Forest modeling with SHAP-based interpretation, microbial association network inference using SparCC and SPIEC-EASI through NetCoMi, correlation heatmaps, and dbRDA/CAP-style association biplots. The platform is implemented as a modular Shiny application so that preprocessing choices are propagated across downstream analyses, results can be exported as figures and tables, and the same application can be run through the public server, source-code installation, or a Docker image.

SimpleMicrobiome consolidates major downstream microbiome analysis tasks in an accessible browser-based environment while retaining links to established analytical frameworks. The platform may reduce technical barriers for non-programming users, improve consistency across exploratory and reporting-oriented analyses, and support collaborative microbiome research. The public application is available at https://simplemicrobiome.mglab.org, the source code is available at https://github.com/yjcho2252/SimpleMicrobiome, and a Docker image for local deployment is available at https://hub.docker.com/r/mglab2252/simplemicrobiome.

## Introduction

High-throughput microbiome profiling is now widely used across biomedical, environmental, agricultural, and translational research. After upstream sequence processing, however, downstream analysis often remains fragmented across multiple packages and scripts, covering sample filtering, taxonomic aggregation, diversity analysis, differential abundance testing, prediction, and microbial network exploration. This fragmentation can increase technical burden, create inconsistencies between analysis steps, and slow collaboration between computational and non-computational researchers.

The R ecosystem provides a mature foundation for microbiome data analysis. Packages such as phyloseq, vegan, ANCOM-BC2, MaAsLin2, randomForest, NetCoMi, ggplot2, and ComplexHeatmap support many of the statistical and visualization procedures commonly used in microbiome studies (Breiman, 2001; Gu et al., 2016; Lin and Peddada, 2020, 2024; Mallick et al., 2021; McMurdie and Holmes, 2013; Oksanen et al., 2026; Peschel et al., 2021; Wickham, 2016). However, combining these tools into a coherent workflow still requires familiarity with data structures, package-specific arguments, transformation choices, filtering strategies, and output handling. Although several graphical platforms, such as MicrobiomeAnalyst 2.0, Mian, Namco, and SHAMAN, have improved accessibility by providing browser-based, no-coding workflows for taxonomic composition and diversity analysis (Dietrich et al., 2022; Jin et al., 2022; Lu et al., 2023; Volant et al., 2020), advanced downstream tasks such as interpretable prediction, two-group network comparison, and taxa-metadata association ordination often remain separated across tools or require additional scripting.

To address this need, we developed SimpleMicrobiome, a modular web application for streamlined downstream microbiome analysis. Rather than introducing a new statistical method, SimpleMicrobiome organizes established analytical approaches into a browser-based workflow designed for accessibility, consistent workflow execution, and exportability.

## Materials and Methods

### Software implementation and application architecture

SimpleMicrobiome is implemented in R using Shiny (Chang et al., 2026). The interface is built with Shiny-compatible packages including bslib, DT, and shinyWidgets, while the analytical back end relies on established microbiome, ecological, statistical, machine-learning, network, and visualization libraries (Perrier et al., 2026; Sievert et al., 2026; Xie et al., 2026). Core dependencies include phyloseq and microbiome for microbiome data handling (Lahti and Shetty, 2017; McMurdie and Holmes, 2013), vegan and cluster for ecological analysis (Maechler et al., 2026; Oksanen et al., 2026), ANCOM-BC2 and MaAsLin2 for differential abundance and association modeling (Lin and Peddada, 2020, 2024; Mallick et al., 2021), the randomForest and shapr R package for predictive modeling and interpretation (Breiman, 2001; Sellereite and Jullum, 2020), NetCoMi, igraph, and ggraph for microbial network inference and graph visualization (Antonov et al., 2023; Pedersen, 2025; Peschel et al., 2021), and ggplot2, ggpubr, ggrepel, ggpattern, and ComplexHeatmap for figure generation (FC et al., 2026; Gu et al., 2016; Kassambara, 2026; Slowikowski, 2026; Wickham, 2016).

The application is organized as task-specific Shiny modules coordinated through a shared internal analysis object. This structure preserves workflow continuity by retaining preprocessing filters and subset choices across downstream analyses.

### Local deployment and reproducible environment

The application can be used through the public web server or installed locally from the source repository. For source-based local installation, the current repository specifies R 4.5.1, the version tested for this release, and provides an install.R script for installing required CRAN, Bioconductor, and GitHub dependencies. To reduce environment-specific installation barriers, we also provide a Docker Hub deployment route based on a pre-built author-maintained image. Users can pull and run the mglab2252/simplemicrobiome:v1.0 image locally without manually compiling the full dependency stack or building an image from the source code.

### Input data and preprocessing

SimpleMicrobiome accepts standard processed microbiome inputs: an abundance table containing ASV, OTU, or feature counts by sample; a taxonomy table aligned to feature identifiers; and a metadata table aligned to sample identifiers. During loading, shared sample and feature identifiers are harmonized, empty entries are removed, and the dataset is prepared for analysis. The current repository includes bundled example datasets for app-level testing and demonstration, including the HMP V3-V5 body-site cohort and SprockettTH multi-age fecal cohort, each provided as a compact subset selected from the original data.

Users can define an analysis-ready subset through minimum read-count filtering, searchable sample selection, metadata-based group toggles, and zero-sum taxon removal. These steps are interactive and reversible within a session, and the resulting processed dataset is used consistently across downstream analyses.

### Analytical workflow

The application interface is organized with a module menu at the top, option and parameter controls on the left, and analysis results with legends on the right (Fig. 1A). The workflow begins with data loading and preprocessing and then proceeds to integrated downstream analysis (Fig. 1B). Taxonomic profiling modules summarize microbial composition across taxonomic ranks and support group-wise comparisons of selected taxa. Diversity modules provide alpha diversity indices including observed richness, Chao1, Shannon, and Simpson diversity, as well as beta diversity workflows with PCoA, NMDS, PERMANOVA, optional clustering, and metadata vector overlays (Anderson, 2001; Oksanen et al., 2026).

For differential abundance and multivariable association analysis, SimpleMicrobiome includes ANCOM-BC2 and MaAsLin2 modules with user-defined grouping variables, covariates, taxonomic ranks, filtering options, and model terms (Lin and Peddada, 2024; Mallick et al., 2021). Where supported by the underlying method, the ANCOM-BC2 and MaAsLin2 modules also allow random-effect or within-subject pairing terms for repeated-measures designs, and a user manual describing module-level options is linked from the main interface. A Random Forest module supports classification or regression with holdout or cross-validation evaluation, permutation importance, ROC/AUC visualization, SHAP-based contribution summaries, and feature ranking views (Breiman, 2001; Jullum et al., 2025; Liaw and Wiener, 2002). Microbial association networks are implemented through SparCC and SPIEC-EASI workflows using NetCoMi (Friedman and Alm, 2012; Kurtz et al., 2015; Peschel et al., 2021). Additional association-level exploration is available through correlation heatmaps and dbRDA/CAP- or CCA-style biplots (Anderson and Willis, 2003; Legendre and Anderson, 1999; Oksanen et al., 2026). Across modules, figures and tabular outputs are downloadable to support manuscript preparation and collaborative review.

### Example dataset preparation

For the example analysis shown in Fig. 2, the Human Microbiome Project V3-V5 16S rRNA gene dataset from Qiita study ID 1928 was processed outside SimpleMicrobiome (Gonzalez et al., 2018; The Human Microbiome Project Consortium, 2012). Sequence data were processed with QIIME 2 and DADA2, taxonomy was assigned using a reference database, and QIIME 2 artifacts were converted into abundance and taxonomy tables using the qza-to-tsv converter at https://simplemicrobiome.mglab.org/convert/ui (Bolyen et al., 2019; Callahan et al., 2016). The metadata table was manually prepared, and a compact subset of oral, skin, and stool samples was selected from the original dataset to provide a clear demonstration of taxonomic composition differences across major body habitats. The repository also includes a second bundled example, SprockettTH_subset, a compact multi-age fecal cohort subset with country and age-related metadata derived from published gut microbiome studies and distributed through microbiomeDataSets (Sprockett et al., 2020; Subramanian et al., 2014; Vatanen et al., 2016). The SprockettTH_subset comprises 120 samples, 1,276 ASVs, and 23 metadata variables, including repeated subject identifiers, delivery mode, feeding status, and multiple age measures. This multi-covariate and repeated-subject structure provides an example dataset for app-level testing and training in multivariable and repeated-measures workflows supported by the relevant modules.

## Results and Discussion

### Example dataset analysis

To demonstrate the workflow on real microbiome data, we analyzed the prepared HMP V3–V5 subset described above. The subset contained 80 samples from six body sites representing oral, skin, and stool habitats: buccal mucosa (n = 13), supragingival plaque (n = 15), tongue dorsum (n = 15), anterior nares (n = 12), right antecubital fossa (n = 11), and stool (n = 14). At the habitat level, the subset included oral (n = 43), skin (n = 23), and stool (n = 14) samples. SimpleMicrobiome reproduced expected habitat-specific structure across modules (Fig. 2). Genus-level composition profiles (Fig. 2A) showed distinct dominant taxa by habitat. Oral samples were enriched for genera such as *Streptococcus*, Veillonella_A, *Prevotella*, Haemophilus_D_735815, and Neisseria_563205, whereas skin samples were characterized by higher relative abundance of *Cutibacterium* and *Corynebacterium*, particularly in anterior nares and antecubital fossa samples. Stool samples showed a different compositional profile, including prominent Bacteroides_H and Phocaeicola_A_858004 signals. These patterns are consistent with the strong niche specialization of microbial communities across human body habitats described in the original HMP study.

Beta diversity analysis further separated samples by body habitat (Fig. 2B). Two-dimensional Bray-Curtis NMDS at the genus level produced clear clustering of oral, skin, and stool samples, with a low stress value of 0.099 in the submitted run, indicating a low-distortion ordination of the underlying distance structure. Vector overlays highlighted habitat-associated gradients such as *Streptococcus* in oral samples and *Cutibacterium* in skin samples. Skin was selected as an illustrative non-oral comparator so that Fig. 2C could highlight oral-associated differential taxa and Fig. 2D could show corresponding oral and skin network examples. ANCOM-BC2 analysis (Fig. 2C) identified genera increased in oral samples, including Neisseria_563205, Veillonella_A, Leptotrichia_A_993758, *Rothia*, *Streptococcus*, Fusobacterium_C, and *Pauljensenia*, together with genera increased in skin samples, including *Corynebacterium* and *Cutibacterium*. SPIEC-EASI network inference generated connected genus-level association networks for oral and skin subsets (Fig. 2D). Together, these outputs illustrate how SimpleMicrobiome can move from composition and ordination to statistical feature ranking and habitat-specific network exploration within a single interface. The four panels in Fig. 2 represent selected examples of the platform's analysis outputs; the same processed dataset can also be examined through other modules, including alpha diversity, PCoA-based beta diversity, MaAsLin2 association modeling, Random Forest prediction with feature interpretation, SparCC network inference, correlation heatmaps, and dbRDA/CAP-style biplots.

To assess numerical concordance between the graphical interface and direct execution of the underlying R packages, we repeated representative analyses of the bundled HMPV3V5 dataset in R using identical input data, preprocessing steps, analysis parameters, and random seeds where applicable. We compared selected module-specific outputs, including alpha-diversity estimates, PERMANOVA statistics, differential-abundance results, Random Forest validation metrics and feature importance, and network results. The GUI and direct R results were concordant at the reported precision; ANCOM-BC2 log-fold changes and MaAsLin2 coefficients matched after applying the two-decimal rounding used for GUI display (Table S1).

### Comparison with representative GUI-based microbiome platforms

We compared SimpleMicrobiome with representative web-accessible platforms, including MicrobiomeAnalyst 2.0, Mian, Namco, and SHAMAN (Table 1) (Dietrich et al., 2022; Jin et al., 2022; Lu et al., 2023; Volant et al., 2020). The comparison focused on selected downstream analysis and implementation-level features that distinguish the platforms beyond commonly available browser-based visualization and diversity-analysis functions.

The compared platforms share several baseline functions, including web-browser access, graphical workflows that reduce coding requirements, taxonomic composition visualization, alpha/beta diversity analysis, and example or manual material. For differential abundance testing and taxa-metadata association modeling, method coverage differs across platforms. SimpleMicrobiome provides ANCOM-BC2 for compositional differential abundance testing and MaAsLin2 for multivariable association modeling, allowing users to evaluate microbial features while considering compositional data structure and metadata covariates (Lin and Peddada, 2024; Mallick et al., 2021). MicrobiomeAnalyst 2.0 provides related compositional and association options, including LinDA and MaAsLin2 (Lu et al., 2023; Zhou et al., 2022), whereas Mian, Namco, and SHAMAN appear to emphasize broader statistical testing, feature selection, regression, correlation, or count-based differential analysis workflows (Dietrich et al., 2022; Jin et al., 2022; Volant et al., 2020). Beyond these differential and association-analysis functions, SimpleMicrobiome integrates Random Forest-based feature ranking with SHAP-oriented interpretation, two-group microbial network comparison using SparCC and SPIEC-EASI workflows, and dbRDA/CAP- or CCA-style ordination biplots for taxa-metadata association visualization (Breiman, 2001; Friedman and Alm, 2012; Jullum et al., 2025; Kurtz et al., 2015; Peschel et al., 2021; Ter Braak, 1986). The platform also combines public web access, open-source availability, Docker-based local deployment, and code-level extensibility through a modular R Shiny source structure. Together, these features position SimpleMicrobiome as a downstream workflow platform for exploratory and reporting-oriented microbiome analysis.

Several limitations should be considered. SimpleMicrobiome is designed for downstream analysis of processed microbiome tables and does not perform raw FASTQ processing. Users must therefore rely on upstream pipelines for quality control, denoising or OTU clustering, taxonomic assignment, and generation of abundance tables. Web-server use also requires attention to data privacy and institutional policies for personal or sensitive metadata, whereas local source-code installation or Docker Hub image deployment may be preferable for restricted datasets. In addition, although the graphical interface reduces programming barriers, it cannot replace expert judgment because appropriate interpretation still depends on study design, sample size, metadata quality, statistical assumptions, and biological domain knowledge. Normalization, filtering thresholds, covariate adjustment, and network parameters should be selected according to study design, sequencing depth, sparsity, sample size, and method. For example, filtering thresholds should be chosen after inspecting sequencing depth, feature prevalence, and zero inflation rather than by applying a fixed universal cutoff. Covariates should be prespecified from the study design or known confounders, and network filtering and edge-retention thresholds should be adjusted according to sample size, feature sparsity, and the exploratory purpose of the analysis. Network edges, Random Forest importance, and SHAP-based feature contributions should be interpreted as exploratory associations or prediction-oriented signals, not as evidence of causal microbial interactions or biological mechanisms. Runtime and memory performance for very large cohorts or highly resolved feature tables should be evaluated further. The current input workflow also does not accept representative sequences or a phylogenetic tree, so tree-dependent diversity metrics such as Faith's phylogenetic diversity and UniFrac distances are not yet available; incorporating optional tree input is a planned improvement. Dedicated longitudinal and repeated-measures workflows, batch-effect detection and correction tools, a one-click reproducible session report, a worked tutorial for non-computational users, and ALDEx2 as an additional compositional differential-abundance method are likewise useful future extensions beyond the current release. Finally, because the compared platforms differ substantially in architecture, deployment model, and intended scope, the present comparison focuses on functional scope rather than formal benchmarking of numerical outputs or usability, which would be difficult to standardize fairly across such heterogeneous tools.

Despite these limitations, SimpleMicrobiome provides a practical and extensible resource for microbiome researchers who need a coherent downstream analysis environment. Its public web access, open-source implementation, bundled example data, Docker-based local deployment, and module-level exports make it suitable for research, collaboration, and training.

## Conclusion

SimpleMicrobiome is an integrated web-based platform for downstream microbiome analysis. By combining data loading, preprocessing, taxonomic profiling, diversity analysis, differential abundance testing, predictive modeling, microbial network inference, and association visualization in a modular R Shiny application, the platform reduces scripting burden while retaining links to established analytical methods. The public server, local source installation, and Docker container support accessible remote and local use by interdisciplinary research teams.

## Figures and Tables

**Fig. 1.**
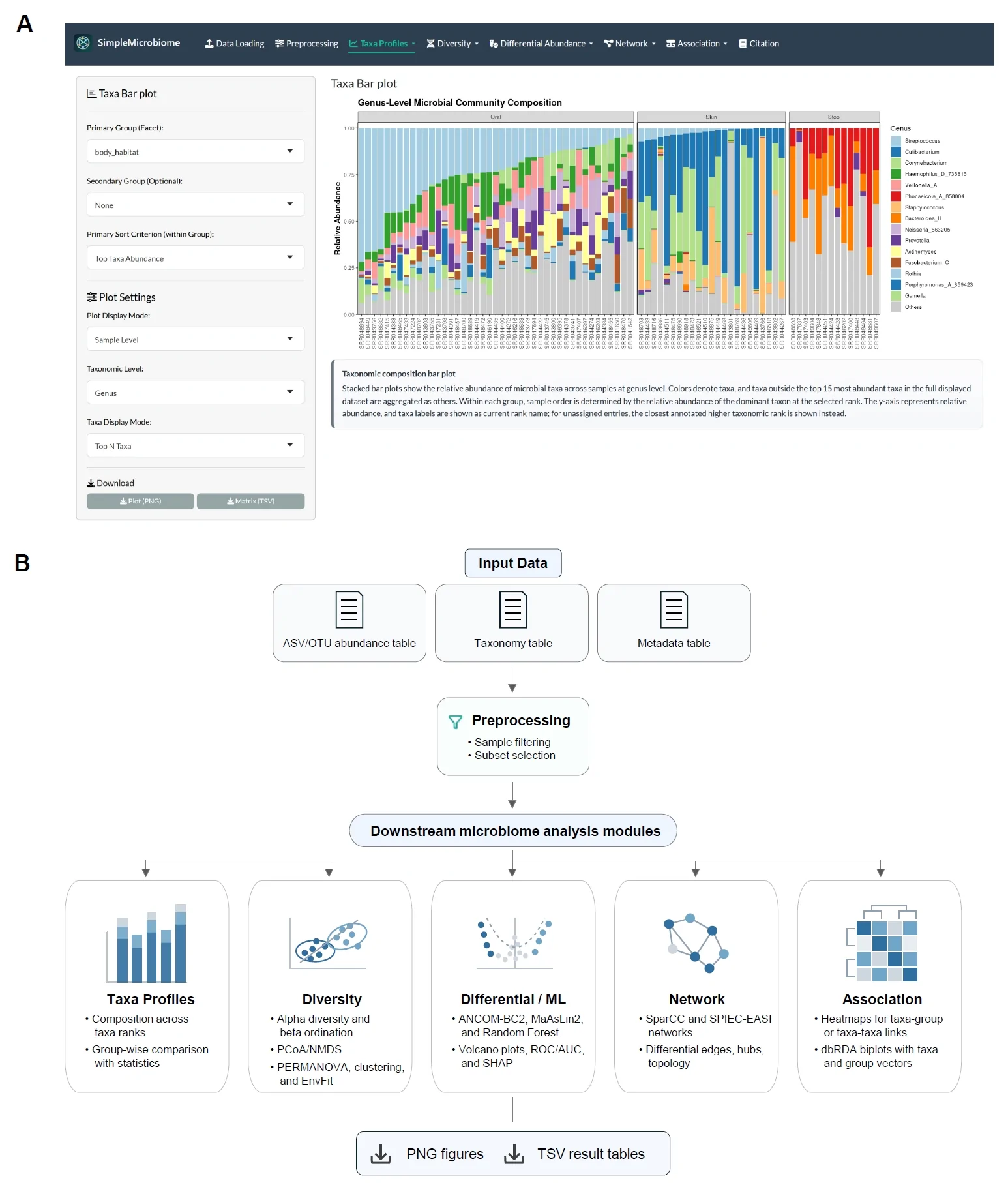
Overview of the SimpleMicrobiome user interface and workflow. (A) Representative screenshot of the Taxa Bar plot module, showing the top navigation bar, module-specific control panel, genus-level taxonomic composition plot, and module description panel. (B) Workflow diagram showing input of processed abundance, taxonomy, and metadata tables, interactive preprocessing and sample filtering, propagation of the processed dataset to downstream modules for taxonomic profiling, diversity analysis, differential abundance testing, predictive modeling, microbial network inference, association visualization, and module-level figure/table export.

**Fig. 2.**
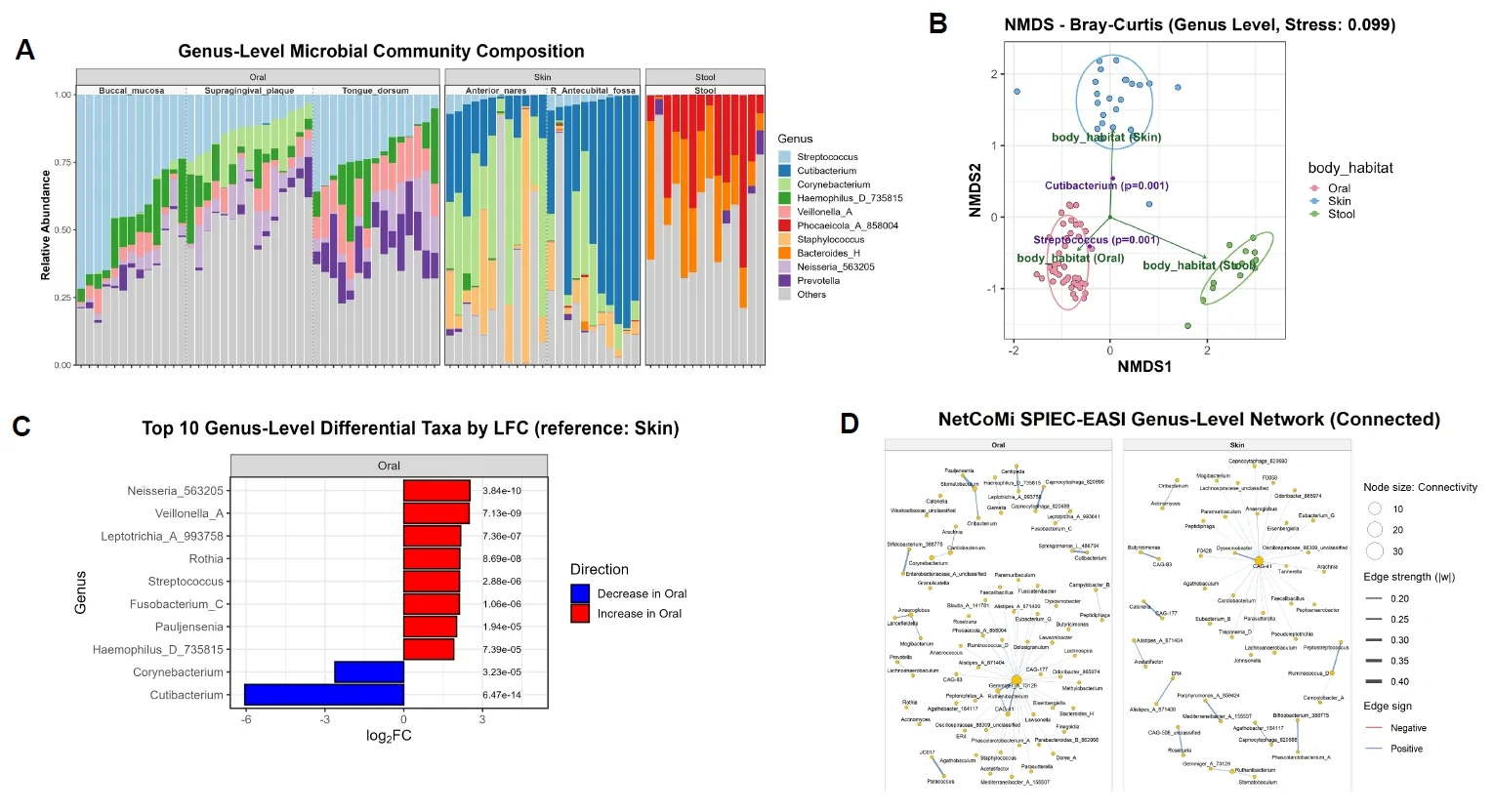
Representative analysis of a Human Microbiome Project example dataset using SimpleMicrobiome. The example dataset was derived from Qiita study ID 1928, a V3-V5 16S rRNA gene dataset from the healthy adult Human Microbiome Project cohort, and includes processed profiles from oral (n = 43), skin (n = 23), and stool (n = 14) body habitats. (A) Genus-level relative abundance profiles grouped by body habitat and body site show habitat-specific community composition across buccal mucosa, supragingival plaque, tongue dorsum, anterior nares, right antecubital fossa, and stool samples. Individual sample names on the x-axis are omitted for readability. (B) Bray-Curtis NMDS ordination at the genus level separates samples by body habitat, with ellipses representing oral, skin, and stool groups and selected taxa or metadata vectors highlighting habitat-associated gradients (dimensions = 2, stress = 0.099, starts = 20, trymax = 20, convergence = best solution repeated 1 time). (C) Top genus-level differential taxa ranked by log_2_ fold change for an illustrative oral-versus-skin ANCOM-BC2 contrast. Red bars indicate taxa increased in oral samples, and blue bars indicate taxa decreased in oral samples relative to skin; q-values are shown beside each taxon. (D) NetCoMi SPIEC-EASI genus-level association networks for oral and skin subsets. Node size represents connectivity, edge width represents association strength, and edge color indicates negative or positive associations.

**Table 1.** Comparison of SimpleMicrobiome and representative GUI-based microbiome analysis platforms. Entries summarize published descriptions, public documentation, repository or source-code information where available, and direct inspection of accessible interfaces. "Yes" indicates clearly documented support, "No" indicates absence or no clear support, and "Partial" indicates narrower or workflow-dependent support.

Feature	SimpleMicrobiome	MicrobiomeAnalyst 2.0	MIAN	Namco	SHAMAN
Raw sequence processing	No	Yes	No	No	Yes
Differential/association modeling	Yes (ANCOM-BC2, MaAsLin2)	Yes (LinDA, MaAsLin2)	Partial	Partial	Partial (DESeq2)
Machine learning feature prioritization	Yes (RF + SHAP)	Yes (RF)	Yes	Yes (RF)	No
Dedicated microbial network inference	Yes (SparCC, SPIEC-EASI)	Yes (SECOM, SparCC)	Partial	Yes	Partial
Taxa-metadata ordination biplots	Yes (dbRDA/CAP, CCA)	Partial	Partial	Partial	Partial
Local/offline deployment from source code	Yes (R Shiny source)	Partial (R package backend)	No	No	Partial (Linux/packrat)
Code-level extensibility	Yes (modular R Shiny)	Partial	No	No	Partial
Docker-based local deployment	Yes	No	No	No	Yes

RF, random forest; dbRDA, distance-based redundancy analysis; CAP, canonical analysis of principal coordinates; CCA, canonical correspondence analysis.

## Supplementary Information

Table S1. Numerical concordance between SimpleMicrobiome GUI outputs and equivalent standalone R analyses using the HMP V3–V5 body-site cohort. All analyses were performed using the default GUI settings. For SparCC and SPIEC-EASI, the “Compare two groups” analysis mode was used. GUI and standalone R outputs were considered numerically concordant when no differences were observed through 12 decimal places. ANCOM-BC2 log fold changes and MaAsLin2 coefficients were compared at the GUI-displayed precision of two decimal places.

## References

[b1-jm-2606011] Anderson MJ (2001). A new method for non‐parametric multivariate analysis of variance. Austral Ecol.

[b2-jm-2606011] Anderson MJ, Willis TJ (2003). Canonical analysis of principal coordinates: A useful method of constrained ordination for ecology. Ecology.

[b3-jm-2606011] Antonov M, Csárdi G, Horvát S, Müller K, Nepusz T (2023). igraph enables fast and robust network analysis across programming languages. arXiv.

[b4-jm-2606011] Bolyen E, Rideout JR, Dillon MR, Bokulich NA, Abnet CC (2019). Reproducible, interactive, scalable and extensible microbiome data science using QIIME 2. Nat Biotechnol.

[b5-jm-2606011] Breiman L (2001). Random Forests. Mach Learn.

[b6-jm-2606011] Callahan BJ, McMurdie PJ, Rosen MJ, Han AW, Johnson AJ (2016). DADA2: High-resolution sample inference from Illumina amplicon data. Nat Methods.

[b7-jm-2606011] https://doi.org/10.32614/CRAN.package.shiny.

[b8-jm-2606011] Dietrich A, Matchado M, Zwiebel M, Ölke B, Lauber M (2022). Namco: A microbiome explorer. Microb Genom.

[b9-jm-2606011] https://doi.org/10.32614/CRAN.package.ggpattern.

[b10-jm-2606011] Friedman J, Alm EJ (2012). Inferring correlation networks from genomic survey data. PLoS Comput Biol.

[b11-jm-2606011] Gonzalez A, Navas-Molina JA, Kosciolek T, McDonald D, Vazquez-Baeza Y (2018). Qiita: Rapid, web-enabled microbiome meta-analysis. Nat Methods.

[b12-jm-2606011] Gu Z, Eils R, Schlesner M (2016). Complex heatmaps reveal patterns and correlations in multidimensional genomic data. Bioinformatics.

[b13-jm-2606011] Jin BT, Xu F, Ng RT, Hogg JC (2022). Mian: Interactive web-based microbiome data table visualization and machine learning platform. Bioinformatics.

[b14-jm-2606011] Jullum M, Olsen LHB, Lachmann J, Redelmeier A (2025). shapr: Explaining machine learning models with conditional Shapley values in R and Python. arXiv.

[b15-jm-2606011] https://doi.org/10.32614/CRAN.package.ggpubr.

[b16-jm-2606011] Kurtz ZD, Müller CL, Miraldi ER, Littman DR, Blaser MJ (2015). Sparse and compositionally robust inference of microbial ecological networks. PLoS Comput Biol.

[b17-jm-2606011] http://microbiome.github.com/microbiome.

[b18-jm-2606011] Legendre P, Anderson MJ (1999). Distance-based redundancy analysis: Testing multispecies responses in multifactorial ecological experiments. Ecol Monogr.

[b19-jm-2606011] Liaw A, Wiener M (2002). Classification and regression by randomForest. R News.

[b20-jm-2606011] Lin H, Peddada SD (2020). Analysis of compositions of microbiomes with bias correction. Nat Commun.

[b21-jm-2606011] Lin H, Peddada SD (2024). Multigroup analysis of compositions of microbiomes with covariate adjustments and repeated measures. Nat Methods.

[b22-jm-2606011] Lu Y, Zhou G, Ewald J, Pang Z, Shiri T (2023). MicrobiomeAnalyst 2.0: Comprehensive statistical, functional and integrative analysis of microbiome data. Nucleic Acids Res.

[b23-jm-2606011] https://doi.org/10.32614/CRAN.package.cluster.

[b24-jm-2606011] Mallick H, Rahnavard A, McIver L, Ma S, Zhang Y (2021). Multivariable association discovery in population-scale meta-omics studies. PLoS Comput Biol.

[b25-jm-2606011] McMurdie PJ, Holmes S (2013). phyloseq: An R package for reproducible interactive analysis and graphics of microbiome census data. PLoS One.

[b26-jm-2606011] https://doi.org/10.32614/CRAN.package.vegan.

[b27-jm-2606011] https://doi.org/10.32614/CRAN.package.ggraph.

[b28-jm-2606011] https://doi.org/10.32614/CRAN.package.shinyWidgets.

[b29-jm-2606011] Peschel S, Müller CL, von Mutius E, Boulesteix AL, Depner M (2021). NetCoMi: Network construction and comparison for microbiome data in R. Brief Bioinform.

[b30-jm-2606011] Sellereite N, Jullum M (2020). shapr: An R-package for explaining machine learning models with dependence-aware Shapley values. J Open Source Softw.

[b31-jm-2606011] https://doi.org/10.32614/CRAN.package.bslib.

[b32-jm-2606011] https://doi.org/10.32614/CRAN.package.ggrepel.

[b33-jm-2606011] Sprockett DD, Martin M, Costello EK, Burns AR, Holmes SP (2020). Microbiota assembly, structure, and dynamics among Tsimane horticulturalists of the Bolivian Amazon. Nat Commun.

[b34-jm-2606011] Subramanian S, Huq S, Yatsunenko T, Haque R, Mahfuz M (2014). Persistent gut microbiota immaturity in malnourished Bangladeshi children. Nature.

[b35-jm-2606011] Ter Braak CJF (1986). Canonical correspondence analysis: A new eigenvector technique for multivariate direct gradient analysis. Ecology.

[b36-jm-2606011] The Human Microbiome Project Consortium (2012). Structure, function and diversity of the healthy human microbiome. Nature.

[b37-jm-2606011] Vatanen T, Kostic AD, d'Hennezel E, Siljander H, Franzosa EA (2016). Variation in microbiome LPS immunogenicity contributes to autoimmunity in humans. Cell.

[b38-jm-2606011] Volant S, Lechat P, Woringer P, Motreff L, Campagne P (2020). SHAMAN: A user-friendly website for metataxonomic analysis from raw reads to statistical analysis. BMC Bioinformatics.

[b40-jm-2606011] https://doi.org/10.32614/CRAN.package.DT.

[b41-jm-2606011] Zhou H, He K, Chen J, Zhang X (2022). LinDA: Linear models for differential abundance analysis of microbiome compositional data. Genome Biol.

