# Supplementary material for: SimpleMicrobiome: An integrated web-based platform for streamlined microbiome data analysis and visualization
Source: J Microbiol. 2026 Sep 18;64(9):e2606011. doi: 10.71150/jm.2606011 (PMC13688125; doi:10.71150/jm.2606011)
Supplement: Supplementary file 1 [file jm-2606011-Supplementary-Table-S1.pdf]

Table S1. Numerical concordance between SimpleMicrobiome GUI outputs and equivalent standalone R analyses using the HMP V3–V5 body-site cohort. All analyses were performed using the default GUI settings. For SparCC and SPIEC-EASI, the “Compare two groups” analysis mode was used. GUI and standalone R outputs were considered numerically concordant when no differences were observed through 12 decimal places. ANCOM-BC2 log fold changes and MaAsLin2 coefficients were compared at the GUI-displayed precision of two decimal places.

<table>
<tr><th>Module</th><th>Compared output</th><th>GUI</th><th>Standalone R script</th></tr>
<tr><td>Taxa bar plot</td><td>Relative abundance</td><td colspan="2">*</td></tr>
<tr><td>Taxa comparison</td><td>CLR-transformed abundance</td><td colspan="2">*</td></tr>
<tr><td>Alpha diversity</td><td>Shannon, Chao1</td><td colspan="2">*</td></tr>
<tr><td rowspan="2">Beta diversity</td><td>PERMANOVA R^2</td><td>0.195</td><td>0.195</td></tr>
<tr><td>PERMANOVA P value</td><td>0.001</td><td>0.001</td></tr>
<tr><td>ANCOM-BC2</td><td>log fold change, P value, q value</td><td colspan="2">*</td></tr>
<tr><td>MaAsLin2</td><td>Coefficient, P value, q value</td><td colspan="2">*</td></tr>
<tr><td>Random forest</td><td>Permutation importance, Accuracy, Macro-F1, One-vs-rest AUC, Confusion matrix</td><td colspan="2">*</td></tr>
<tr><td>SparCC</td><td>Edge identities, Edge weights</td><td colspan="2">*</td></tr>
<tr><td>SPIEC-EASI</td><td>Edge identities, Edge weights</td><td colspan="2">*</td></tr>
<tr><td>Correlation heatmap</td><td>Taxon-by-group association values</td><td colspan="2">*</td></tr>
<tr><td rowspan="2">Association biplot</td><td>PERMANOVA R^2</td><td>0.546</td><td>0.546</td></tr>
<tr><td>PERMANOVA P value</td><td>0.001</td><td>0.001</td></tr>
</table>

*Taxon-specific numerical values differed among taxa but were concordant between the GUI and standalone R execution for all evaluated taxa at the reported precision.
